# Sex Differences in Cardiovascular Disease Risk Among Chinese Older Adults Living with HIV

**DOI:** 10.1093/geroni/igaf122.1612

**Published:** 2025-12-31

**Authors:** Junwen Yu, Bei Wu, Xiang Qi, Lu Shao, Ting Xu, Hongzhou Lu, Zheng Zhu

**Affiliations:** Fudan University, Shanghai, Shanghai, China (People’s Republic); NYU Shanghai, Shanghai, China (People’s Republic); New York University, New York, New York, United States; University of Hong Kong, Guangzhou, Guangdong, China (People’s Republic); Nanjing Medical University, Nanjing, Jiangsu, China (People’s Republic); The Third People’s Hospital of Shenzhen, Shenzhen, Guangdong, China (People’s Republic); Fudan University, Shanghai, Shanghai, China (People’s Republic)

## Abstract

People living with HIV have twice the risk of developing cardiovascular disease (CVD) compared to HIV-negative individuals, yet sex differences remains unclear. This study aimed to address this gap by examining sex differences in CVD risk among Chinese older adults living with HIV. We analyzed data from hospitalized HIV-infected individuals aged 50 years or older at a major hospital in Shenzhen, China, from 2017 to 2022. The 10-year CVD risk was estimated using the Pooled Cohort Equations. Logistic regression and marginal analyses were performed, and a segmented regression model was used to analyze the relationship between high CVD risk prevalence and age, grouped into 5-year intervals. Among the 576 participants, 214 (37.2%) had high CVD risk (9.6% in females and 43.9% in males). The average CVD risk was significantly higher in males than females (8.79% vs. 3.58%, P < 0.001). High CVD risk was significantly associated with CD4 counts < 200 cells/μL (OR = 1.832, 95% CI = 1.101-3.050, P = 0.020). The predicted probability of low CD4 counts was higher in males (46.8%) than females (35.5%, P = 0.016). The prevalence of high CVD risk increased significantly in males after age 50, whereas in females, the breakpoint occurred at age 65. Males exhibited a significantly higher CVD risk, emphasizing the need for early monitoring from a younger age. Although females demonstrated a later onset of high CVD risk, menopause-related lipid metabolism changes highlight the need of early prevention and integrating female-specific factors into risk assessment tools. Future research should further investigate the underlying mechanisms to improve precise prevention strategies.

